# Analysis of the Effects of an Unconventional Rolling Process under Cumulative Plastic Deformation Conditions

**DOI:** 10.3390/ma16093352

**Published:** 2023-04-25

**Authors:** Jacek Pawlicki, Adam Płachta

**Affiliations:** 1 Department of Railway Transport, Faculty of Transport and Aviation Engineering, Silesian University of Technology, Krasińskiego 8, 40-019 Katowice, Poland; 2 Department of Production Engineering, Faculty of Materials Engineering, Silesian University of Technology, Krasińskiego 8, 40-019 Katowice, Poland

**Keywords:** rolling, copper alloys, equivalent strain, shear deformation, unconventional method, metal forming, severe plastic deformation

## Abstract

This article presents the results of research carried out on an experimental rolling mill with axial, cyclic movement of rolls (RCMR). The device was made on the basis of an unconventional technical solution for the movement of shaping tools and equipped with a complete measuring system recording all the parameters of the process. The research was conducted on selected copper alloys CuFe2 and CuCr0.6. Rolling tests in the RCMR process were carried out for rolling speeds vr = 3.1 × 10^−3^, 6.3 × 10^−3^ and 9.4 × 10^−3^ m/s, which correspond to the rotational speed of the rollers at ω = 1, 2 and 3 rpm for an active diameter of the rollers = 60 mm. In testing the thermal effects of the process, the rolling speed ω = 0.7 rpm was also used. A constant value of the frequency of axial movement of the rollers f = 1 Hz and the amplitude of the displacement of the rollers A = 0.8 mm were assumed. The rolling process for the strands was carried out in six culverts using the average relative crush in the passage of Δh = 15%. Conventional rolling tests were carried out to compare rolling processes, and the obtained data formed the basis for assessing the strain intensity and identifying local deformation zones in the RCMR rolling process. The waveforms of rolling pressures, intensity and non-uniformity of deformation, and increase in the temperature of the strip surface in subsequent culverts were compared with the results obtained in the conventional rolling process.

## 1. Introduction

The commonly used methods for large plastic deformation SPD (Severe Plastic Deformation), despite their great research capabilities, have significant limitations, resulting from imperfections in the construction of stations and the lack of appropriate control and recording systems for monitoring the course of these complex deformation processes. Our own experience, gained during the construction of laboratory stations, confirms that, in many deformation processes, it is necessary to program the deformation path in the control system in advance, which of course further complicates the construction of devices [1,2,3]. Another important problem in the analysis of the results of complex shaping processes, most often multi-stage, is the use of an appropriate computational procedure, as well as the awareness of phenomena accompanying the subsequent stages of deformation. This applies both to the correctness in determining the value of a single deformation, total equivalent deformation (cumulative deformation), assessment of deformation unevenness, and selection of a representative place for microstructural analysis. In these processes, the analysis of phenomena differs significantly from the commonly known conventional processes [4,5,6]. The lack of necessary experimental knowledge gained directly during research and the correct use of constitutive equations implies certain negative methodological effects, e.g., determining a reliable value for the total equivalent strain in the process; thus, the results of analyzes of structural effects caused by high plastic deformation may raise doubts.

The basic issue in assessing the correctness of the course of these processes is the ongoing monitoring of the process due to the loss of material coherence, location of the deformation, incorrect material guidance in the deformation valley, temporary lack of contact between the material and the tool surface during deformation, etc. Often, the impact of additional effects generated by the process conditions, in the form of friction, e.g., in the ECAP (Equal Channel Angular Pressing) jacking test, or the thermal phenomena associated with high deformation intensity, are so large that they completely take over the deformation process [7,8,9,10,11,12,13,14]. The equipment used allows for partial monitoring of these phenomena, e.g., thermal imaging [15]. However, in most SPD methods, the possibilities of tracking these phenomena on an ongoing basis are very limited due to difficult access to the deformation zone.

This paper presents the results of tests carried out on a prototype experimental rolling mill, which may be an interesting alternative to the commonly known methods used for large plastic deformations. The tests were carried out on selected CuFe2 and CuCr0.6 copper alloys. These are wrought alloy copper grades containing less than 2% of the main alloying element. Chromium copper is characterized by low production costs, but also has the highest electrical conductivity among copper alloys. The chromium content is in the range 0.4–1.2%, and these alloys are used in the production of, e.g., welder electrodes. Copper–iron alloy with 1.8–2.6% Fe content is characterized by good electrical conductivity. The most frequently produced semi-finished products made of ferrous copper are tapes, which are used to produce elastic elements, trace holes for integrated circuits, connectors, housings for electrical devices, construction elements for computers and mobile phones. Both alloys are subjected to heat treatment, most often solution and aging, in order to give them better strength properties.

The main objective of this experimental research was to compare the conventional rolling process with the Rolling with Cyclic Movement of Rolls (RCMR), by assessing the mechanical, structural and thermal effects.

## 2. Research Stand and Methodology

**Figure 1:**
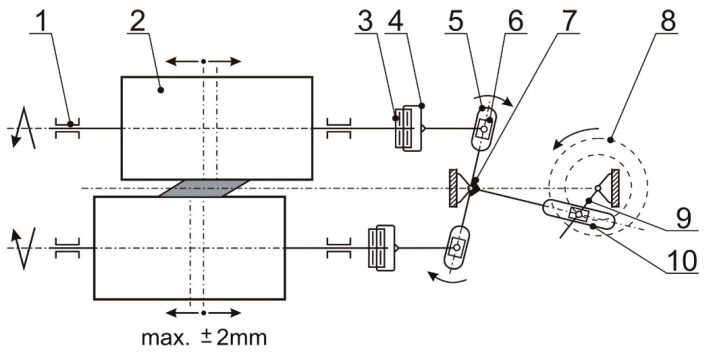
Scheme of RCMR set up. 1. Bearing 2. Working roll 3. Thrust bearing 4. Hushing 5. Yoke 6. Stone sliding 7. Swivel 8. Eccentric bush 9. Eccentric shaft. 10. Stone sliding [16,17].

The station for rolling in the RCMR process was designed as a duo-roll system. The mechanism of the axial movement of the rolls is an original design solution for the experimental rolling mill [16,17]. A rolling process carried out in this way is characterized by the possibility of obtaining much higher values of effective deformation. The RCMR process differs from conventional rolling by the additional lateral movement of the rolls. The strand is deformed by simultaneous height reduction and forced displacement of the material layers in a direction perpendicular to the main direction of rolling. In the conventional rolling process, there is a reduction in the strand height (crease), and the flow of the material in the direction perpendicular to the direction of rolling (widening) is negligible. Figure 1 shows a kinematic diagram of the device used in the RCMR process.

The implementation of the axial movement of the rollers is forced by the hype yoke 5, enforced by a pendulum built on pivots 7, which alternately affects the working rollers 2, moving them along their axis. Yoke 5 is set in motion by an eccentric system consisting of eccentric shaft 9 and eccentric sleeve 8, which cyclically move yoke 5. This cyclic movement of yoke 5 is transmitted to the housings of four bearings 3, and through a bidirectional thrust bearing 3 to working shafts 2. The amount of axial movement of working rollers 2 is regulated by changing the position of eccentric sleeve 8 on eccentric shaft 9. Eccentric sleeve 8 is adjustable relative to eccentric shaft 9 as a result, whereby the resultant eccentricity can vary from zero to the sum of partial eccentricities. This allows for smooth adjustment of yoke deflection 5 and, thus, smooth and stepless axial travel of the working rollers 2.

**Figure 2:**
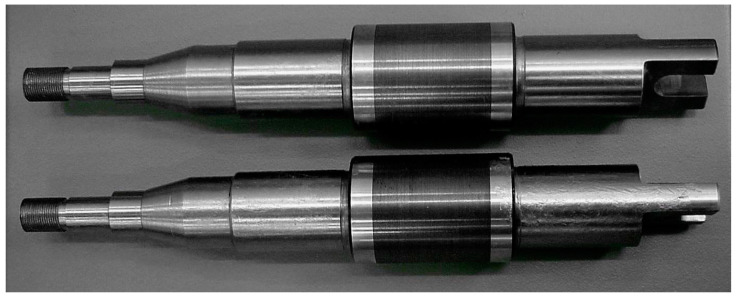
Rolls with parallel grooves on the surface of the barrels.

The design of the device allows quick exchange of rollers, setting the gap between the rollers and the desired size of the axial stroke. Adjustments and settings of the device allow change in the deflection of the cylinder from the middle position to ±2 mm, and the frequency of lateral fluctuations of the rollers by up to 3 Hz. Transverse deformation of the band is forced by parallel grooves made on the surfaces of roller barrels (Figure 2). The device is equipped with a measuring system from BMCM, Germany. The measurement system is controlled, processed and stored using the NEXT VIEW 3.4 program.

The tests were conducted on CuFe2 alloys (2% wt. Fe) and CuCr0.6 (0.6 wt. Cr). The materials were heat treated and supersaturated at 1000 °C/1 h/water. Samples (bands) in the form of flat bars with a square cross-section of 8 × 8 mm and a length of l0 = 60 mm were used. Deformation tests in the RCMR process were carried out for rolling speeds v_r_ = 3.1 × 10^−3^, 6.3 × 10^−3^ and 9.4 × 10^−3^ m/s, which correspond to the rotational speed of the rolls ω = 1, 2 and 3 rpm for an active diameter of the rolls ϕ = 60 mm. In the tests of the thermal effects of the process, the rolling speed ω = 0.7 rpm was also used. A constant value of the frequency of axial movement of the rollers f = 1 Hz and the amplitude of the displacement of the rollers A = 0.8 mm were assumed. The deformation process was carried out in six culverts using the average relative crush in the passage, ε_h_ = 15%. Conventional rolling tests were carried out to compare processes, and the obtained data formed the basis for assessing the strain intensity and identifying local deformation zones in the RCMR rolling process.

Total equivalent deformation in the RCMR process was determined from the relationship:

(1) $$\varepsilon_{st}=\sum_{i=1}^{n}\sqrt{\varepsilon_{hi}^{2}+\varepsilon_{si}^{2}}$$

(2) $$\varepsilon_{hi}=\ln\frac{h_{1}}{h_{i-1}}$$

(3) $$\varepsilon_{si}=\frac{2A}{\sqrt{3}(h_{i-1}+h_{i})}$$

where: ε_et_—total equivalent deformation, ε_hi_—deformation caused by height reduction (actual crease), ε_si_—shear deformation caused by the transverse displacement of the cylinders, n—number of single deformations (number of penetrations, h_i−1_, h_i_—height of the sample (band) before and after a single deformation (culvert), and A—amplitude of the transverse displacement of the cylinders.

In the process of conventional rolling, the form deformation caused by the transverse displacement of the rolls ε_si_ = 0 (Equation (1)), and the determination of the total deformation in the process, was reduced to the determination of the total real crush ε_ht_ for n culverts from the relationship:

(4) $$\varepsilon_{st}=\varepsilon_{ht}=\sum_{i=1}^{n}\varepsilon_{hi}$$

## 3. Kinematic Analysis of the Mechanism of Axial Movement of Rollers

When rolling on the duo rolling machine of the free band (without tension and counter-thrust), with the established parameters and the assumption that the rolled strip does not show deviations in shape in the longitudinal and transverse direction, the rolled strip is characterized by isotropy of plastic properties and is arranged centrally in the axis of the cage, and the rollers do not show shape errors (a constant size of the gap is maintained) in the system of forces acting between the rolled strip and the rolls and, further, between the rolls and others The elements of the rolling cage are fixed.

**Figure 3:**
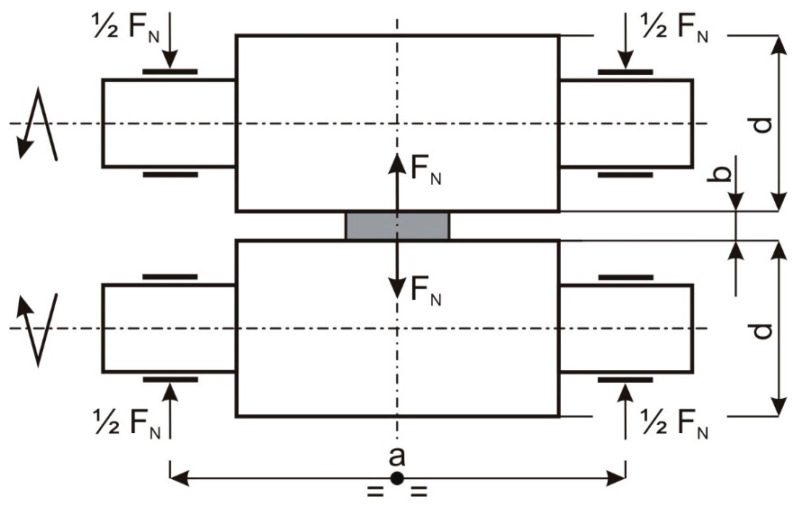
Distribution of forces on roll pins.

It can be assumed that the rolling pressure is divided in half, into the left and right journals of the roller, and if we also do not take into account the internal masses of the bearing arrangements (the weight of the roller including bearings and bearing housings), the forces acting on the left and right journals of the upper and lower cylinders are equal. This is shown in Figure 3. In addition, the tipper moment acting on the cage is equal to zero and therefore the foundation bolts of the cage are loaded only with preload.

The state described above will change if the rollers, in addition to the rotational motion, perform oscillating axial movements, where they are opposite to each other and there is no slippage between the rollers and the band.

**Figure 4:**
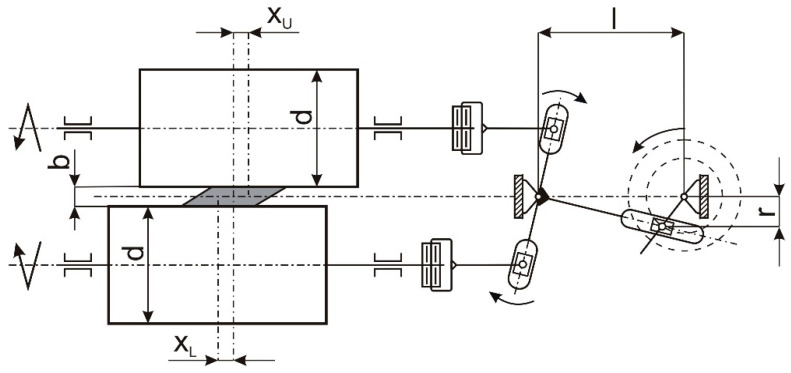
Geometric parameters of the mechanism of axial movement rollers.

Regardless of the type of mechanism causing the axial movement of the rollers and the way it is related to the cage, a tipping moment will appear on the cage, the plane of action of which coincides with the plane containing the roller axes. Therefore, it is a moment acting perpendicular to the subversive moment also acting on the cage in the current method of rolling the strand (without axial movement). The basic geometrical parameters of the mechanism of the axial movement of the rollers are shown in Figure 4.

The kinematic analysis of the mechanism shown in Figure 4 shows that the axial displacements of the upper cylinder x_U_ and lower cylinder x_L_ are:

(5) $$x_{U}=-x_{L}=\left(\frac{d}{2}+\frac{b}{2}\right)\frac{r\sin\phi }{l+r\cos\phi }$$

Assuming that d >> b and l >> r, Formula (5) takes the simple form:

(6) $$x_{U}=-x_{L}=\frac{dr}{2l}\sin\phi$$

The axial displacements of the x_U_ and x_L_ rollers are determined from Formula (6) and the velocities of the rolls in axial motion determined as:

(7) $$v_{U}=\frac{dx_{U}}{dt}=\frac{dr}{2l}\cos\phi$$

(8) $$v_{L}=\frac{dx_{L}}{dt}=-\frac{dr}{2l}\cos\phi$$

**Figure 5:**
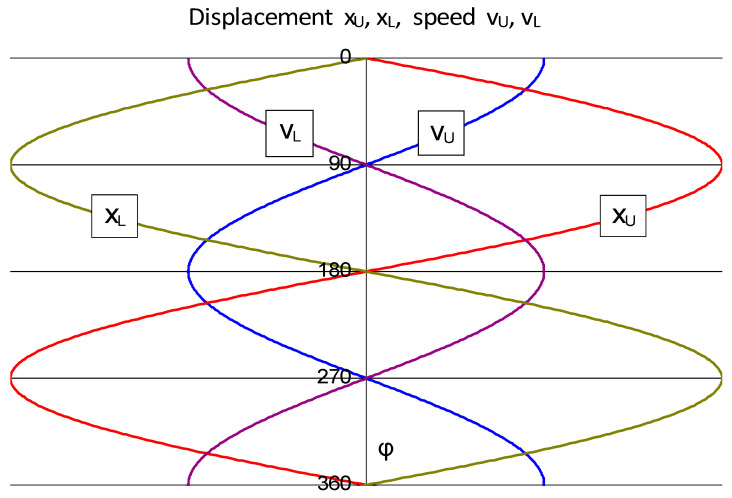
Axial displacement of x_U_ and x_L_ rolls and v_U_ and v_L_ roll speeds in axial movement.

In the graph shown in Figure 5, the following characteristic points can be distinguished:

- The rollers are located in the central positions:
  - axial displacement of the upper cylinder x_U_ = 0,
  - axial displacement of the lower cylinder x_L_ = 0,
  - the velocities of the cylinders in axial motion are directed oppositely and reach maximum values v_U_ = −v_L_ = v_max_.
- The rollers are in opposite extreme positions:
  - axial displacement of the upper and lower cylinder xU = −xL,
  - velocities of rollers in axial motion vU = vL = 0.

Knowledge of the course of displacement and of the velocity of the rollers in axial motion is important, because these quantities describe the parameters of the form deformation of the band in the contact zone with the rollers. These parameters are the magnitude and speed of the form deformation. It can be stated that the force necessary to induce the character deformation, and thus the axial force to be applied to the cylinder, consists of two parts:

- the first depends on the size of the form deformation, i.e., on the axial displacement of the cylinder,
- the second depends on the speed of deformation, i.e., on the speed of movement of the axial cylinder.

**Figure 6:**
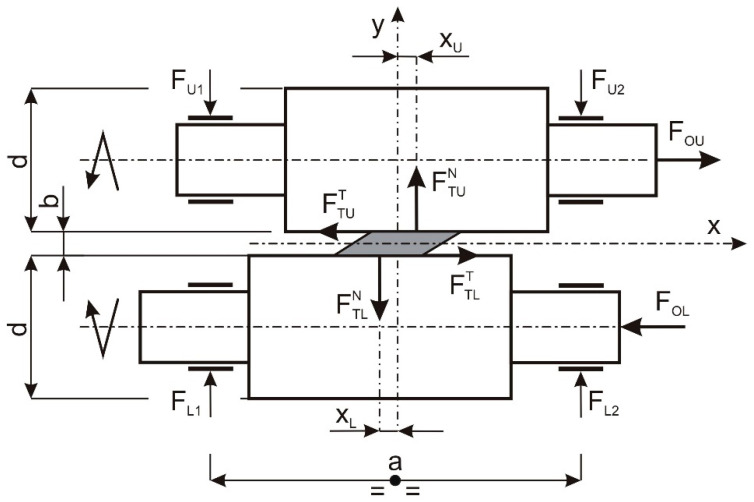
Distribution of forces acting on rollers in the rolling process with cyclic, axial movement of the rolls.

Disregarding the dependencies between the parameters of the axial movement of the rolls and the forces necessary to induce them, it can be stated, in the case of the process with cyclic, axial movement of the rolls, in the system of forces acting between the rolled strip and the rolls, as well as between the rolls and the elements of the mechanism forcing the axial motion of the rolls, there will be two pairs of forces. The resulting two pairs of forces will result in oscillatory differentiation of the forces acting on the roll necks, bearings, rolling gap setting elements, stands, etc. The appropriate diagram of the system of forces acting on the rolls is shown in Figure 6.

The equilibrium conditions for the upper cylinder are as follows:

(9) $$\sum F_{ix}=F_{OU}-F_{TU}^{T}=0$$

(10) $$\sum F_{iy}=-F_{U1}-F_{U2}+F_{TU}^{N}=0$$

(11) $$\sum M_{io}=\frac{a}{2}\left(-F_{U1}+F_{U2}\right)+\left(\frac{d}{2}+\frac{b}{2}\right)F_{OU}-\frac{b}{2}F_{TU}^{T}-x_{U}F_{TU}^{N}=0$$

and accordingly for the lower cylinder:

(12) $$\sum F_{ix}=F_{OL}-F_{TL}^{T}=0$$

(13) $$\sum F_{iy}=F_{L1}-F_{L2}+F_{TL}^{N}=0$$

(14) $$\sum M_{io}=\frac{a}{2}\left(F_{L1}-F_{L2}\right)+\left(\frac{d}{2}+\frac{b}{2}\right)F_{OL}-\frac{b}{2}F_{TL}^{T}-x_{L}F_{TL}^{N}=0$$

Using Dependencies (9) and (10), Dependence (11) can be presented in the following form:

(15) $$\frac{a}{2}\left(-F_{U1}+F_{U2}\right)-x_{U}\left(F_{U1}+F_{U2}\right)+\frac{d}{2}F_{OU}=0$$

and using dependencies (12) and (13), dependence (14) can be presented in the form:

(16) $$\frac{a}{2}\left(F_{L1}-F_{L2}\right)-x_{L}\left(F_{L1}+F_{L2}\right)+\frac{d}{2}F_{OL}=0$$

(15) and (16) show the relationships between the forces on the cylinder journals, its axial displacement and the axial force applied to the journal.

## 4. Process Flow and Efficiency

**Figure 7:**
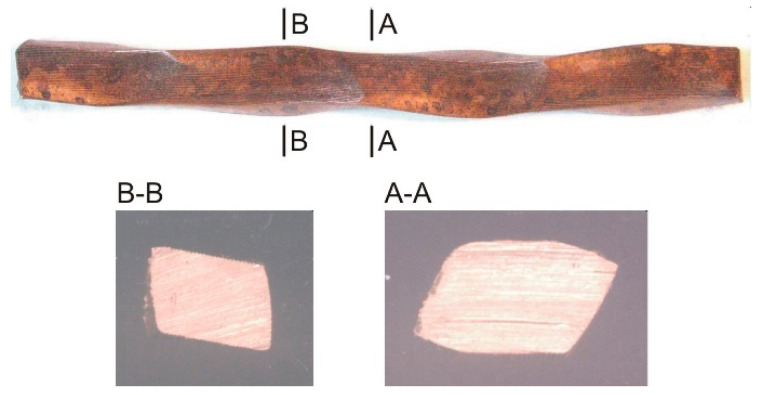
The upper view of a rolled material and cross sections after 1 pass of rolling with the change of loading scheme. Process parameters: ε_h_ = 0.2, ω = 20 rpm, f = 1 Hz, A = 4 mm.

The basic issue in the analysis of the RCMR rolling process is the behavior of the band during rolling. The burden of kinematic parameters of rolling which are too extreme, and above all the high amplitude and frequency of axial movement of the rolls, causes the occurrence of high axial forces and may result in rotation of the band, instead of deformation of the material in the transverse direction to the direction of rolling. This negatively affects the course of the process, the surface condition of the band material, the amount of actual cumulative deformation, and causes an undesirable effect of distortion of the cross-section of the band (Figure 7).

The type of material and the condition after heat treatment also affect the course of the process. Metallic materials with high ductility can be rolled with much higher kinematic process parameters. Thus, we obtain higher values of cumulative deformations. Materials with low ductility require great care in the selection of the most advantageous rolling parameters. This is due to the need to maintain constant contact of the material with the surface of the tool in subsequent phases of the transverse movement of the rollers.

The stable course of the RCMR rolling process, in addition to the kinematic parameters, depends on many variables, especially type of material, geometry of the band and tools, and the shape and size of cuts on the surface of roller barrels. The most beneficial effects of the rolling process can be obtained in the case of a selection of process parameters that will ensure the maximum number of cycles of change of deformation path during the passage of the material through the rolling basin. This is possible if the rolling speed and amplitude of roll deflection, and the maximum frequency of axial movement of the rollers, are used. Discretionary selection of process parameters, not supported by research experience gained directly on the test stand during rolling attempts, is inappropriate, leading to erroneous conclusions and unjustified opinions regarding the test method.

**Figure 8:**
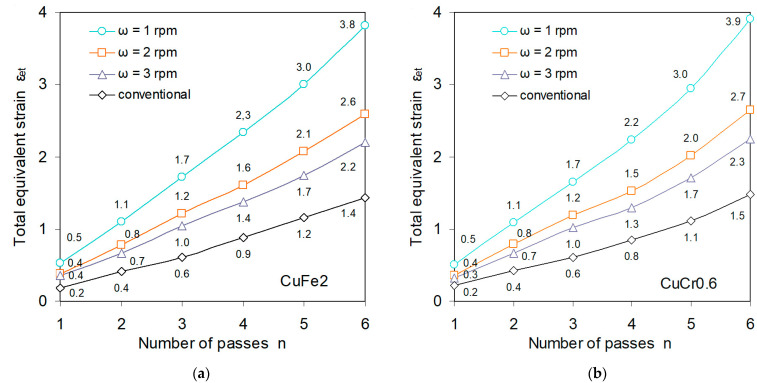
Influence of rolling speed on the value of total equivalent strain for CuFe2 (**a**) and CuCr0.6 (**b**) depending on the number of passes n, with a constant displacement amplitude for transverse rollers A and frequency f.

The measure of the efficiency and effectiveness of the new rolling process is the amount of total deformation obtained. The amount of total equivalent deformation in the RCMR process is influenced by the kinematic parameters of the process and the number of individual deformations, i.e., the number of bushings. Figure 8 shows the influence of the number of culverts on the total strain value in conventional rolling and RCMR. The obtained value of total deformation after six bushings in the RCMR process is about 2.5 times higher than that obtained in the conventional rolling process. This result was obtained for the rotational speed of the rollers ω = 1 rpm. The values of the total equivalent strain for speeds ω = 2 and 3 rpm are correspondingly lower. This regularity results from a decrease in the number of axial movements of rolls along the length of the rolling basin with an increase in the rotational speed of the rolls (rolling speed). The value of total equivalent deformation in the RCMR process is defined as the average value of deformation over the length of the rolling basin.

The effectiveness of the method is also evidenced by the distribution of deformations in the section of the rolled strip. A qualitative analysis of the evolution of local deformation zones in the RCMR process was made on the basis of structural studies of the cross-sections of the bands in subsequent passes to obtain the maximum value of total deformation. The maximum value of the total deformation was determined by the technical capabilities of the device and the efficiency of transferring the lateral movement of the rolls to the deformed material. For comparison, conventional rolling tests were carried out with the same values of single strain ε_hi_ in a pass, and total strain ε_ht_ after n passes.

**Figure 9:**
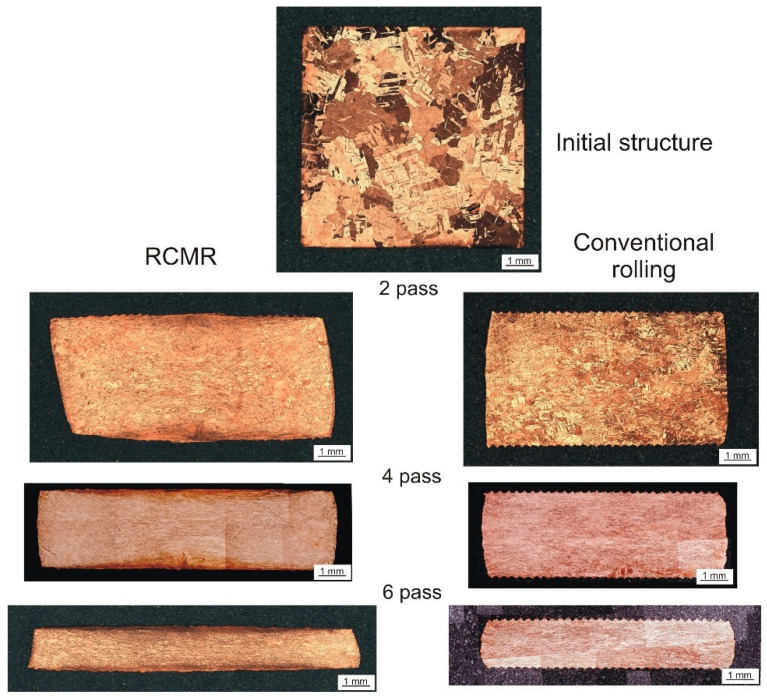
Microstructural images of sample cross sections in selected passes in the RCMR and conventional rolling process (rolling speed ω = 1 rpm).

Observations of the cross-sectional structure of the bands in subsequent culverts in the RCMR process indicate a significant influence of additional transverse roll movement on the evolution of local deformation zones. Figure 9 shows microstructural images of cross-sections of CuFe2 alloy samples for selected culverts. The transverse, cyclic movement of the rollers already causes in the initial phase of deformation (in the initial culverts) the formation of strongly deformed zones at the contact surfaces with the upper and lower cylinder. The near-surface intensively deformed zones migrate in successive culverts from the surface towards the center of the bandwidth height and cover an increasing volume of the band. The effectiveness of the impact of rollers on the size and location of zones of large plastic deformation increases with the number of culverts.

In the middle zone of the strand cross-section, we observe a structure that is similar to that obtained in the conventional rolling process. In this zone, the deformation is mainly caused by linear deformation related to the reduction of the sample height (Δh_i_ = h_0_ − h_i_, (Δh_i_—reduction of the sample height after the i-th pass, h_0_—the initial height of the sample, h_i_—the height of the sample after the i-th pass)). It follows that the additional impact associated with the cyclic transverse movement of the rolls decreases with increasing distance from the contact surface of the material and the tool to the center of the band height.

Significant differences were also observed in the geometry of the cross-sections of the rolled strands. The cross-sections of the strips in the RCMR process are characterized by a greater width (increase in the broadening Δb) compared to the corresponding cross-sections of conventionally rolled strips. This proves the high effectiveness of the impact of the transverse movement of the rollers. In the conventional rolling process, due to the hindered strand flow in the width direction caused by cuts on the roll barrel surfaces, the strand widening is insignificant, intensifying the plastic flow of the strand in the rolling direction. An additional observed geometric effect of the cross-sections of strips rolled using the RCMR method is the characteristic shape of the cross-section, similar to a parallelogram, particularly visible in the initial culverts and disappearing as the number of culverts increases. The analysis of the geometry of the cross-sections of rolled strands is important from the point of view of the final rolled product. Taking up this issue is necessary in undertakings aimed at commercialization of the presented method of rolling.

Much more intensive plasticization of the material in the RCMR process, affecting the geometry of the strand cross-section, is also evidenced by the actual height of the rolled strand corresponding to the given pass (with the same setting of the rolling gap). In the process, the RCMR is noticeably lower compared to conventional rolling, which was confirmed by the measurements.

The effect of deformation softening in the RCMR rolling process is caused by a permanent change in the deformation path. This phenomenon has already been observed in laboratory tests and technological processes, where the effect of changing the orientation of the main stress components is revealed by a decrease in the hardness of materials in the area of the center of the deformation basin. The phenomenal analysis of the specific mechanism of plastic deformation of metals under the influence of external conditions, as a result of cyclical forcing of a change in the deformation path, was carried out by Korbel [18,19,20]. According to this original concept, the essence of the mechanism of superplastic flow of metals are two synchronized phenomena: the location of deformation in the shear bands and the recrystallization of the band areas, involving more and more new fragments of material. The validity of the concept proposed by Korbel was confirmed by experimental studies stretching mono and polycrystalline copper [21,22]. The research focused on determining the causes and understanding the effects of self-excitation and forced change of the deformation path, regarding the possibility of controlling the structure of the deformed metal, and thus its mechanical properties. The concept allowed the development of an original method of extrusion of KoBo metals with a cyclic torsion matrix [23,24,25,26].

**Figure 10:**
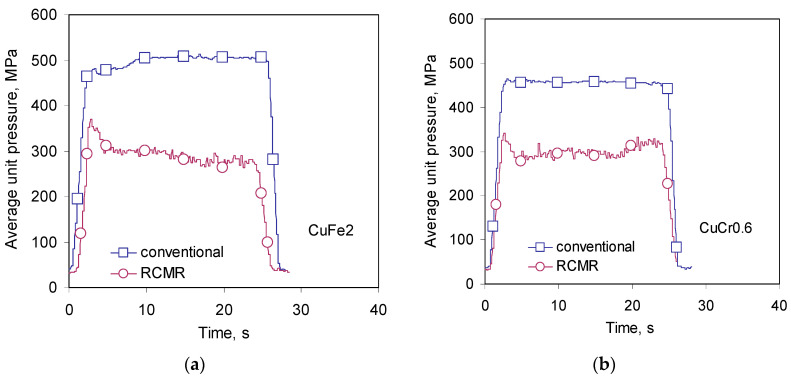
Characteristics of average unit pressures of rolling in the 5th pass for CuFe2 (**a**) and CuCr0.6 (**b**). Rolling speeds ω = 1 rpm.

The effect of deformation softening is also noticeable in the course of unit rolling pressures. Figure 10 shows examples of average unit loads of rolling in the RCMR process and conventional rolling. The level of pressure in the RCMR process in subsequent culverts is significantly lower compared to conventional rolling. The deformation softening effect occurs for both rolled materials and is independent of the type of material.

## 5. Thermal Effect in the RCMR Process

Force-energy and microstructural phenomena, accompanying the new deformation method in the rolling process, are also thermally activated. Local temperature rises in microvolumes of an intensively deformed material can reach the starting temperature of recrystallization of a given material. In microstructural studies, the appearance of germs of new grains was observed within a strongly deformed matrix along the shear bands. Shear bands are particularly privileged places for the initiation and development of recrystallization. The volume proportion of these areas in relation to the total volume of deformed material is insignificant. It can be assumed that the appearance of recrystallization embryos in the areas of shear bands is a secondary process to the location of deformation in the shear bands. The assessment of the impact of these phenomena, caused by high plastic deformation, on the effect of increasing material plasticity and decreasing shaping pressures requires further analysis.

However, the observed increase in the temperature of the deformed material measured on the band surface, even by several dozen degrees, affects the force and energy parameters of the process, especially for non-ferrous metal alloys with appropriately lower temperatures of structure renewal, healing and recrystallization. The unconventional rolling process is characterized by high deformation intensity, and the shear deformation rates resulting from the transverse movement of the rolls are significant. The increase in the band temperature is caused by two simultaneous phenomena: friction in the contact zone of the material and the tool enabling the material to be moved in the direction perpendicular to the rolling direction, and the conversion of part of the work of plastic deformation into thermal energy.

The analysis of temperature fields uses a FLIR A320 thermal imaging camera and ThermaCAM Researcher Pro version 2.8 thermographic image processing software. The advantage of measuring the temperature of the deformed surface of the sample based on the detection of infrared radiation is the ability also to control the uniformity of deformation in the rolling mill basin and, what is important, the method is inertia-free. However, accurate thermographic assessment of thermal phenomena in the rolling process is difficult due to significantly limited access to the space of the deformation basin. In the well-known methods using large plastic deformations, access to strongly deformed zones is also difficult due to the construction of the devices and the shape of the tools.

**Figure 11:**
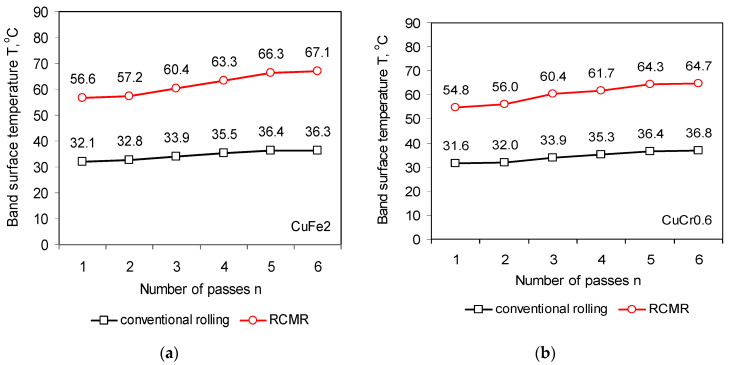
Band surface temperature in the process of conventional rolling and RCMR of CuFe2 (**a**) and CuCr0.6 (**b**) alloys at a rolling speed ω = 1 rpm.

The analysis of the obtained thermographic images showed an intense increase in the strip surface temperature in the rolling process with cyclic, axial movement of the rolls in comparison to conventional rolling. Figure 11 shows the band surface temperatures in successive passes in the conventional and RCMR rolling process.

**Figure 12:**
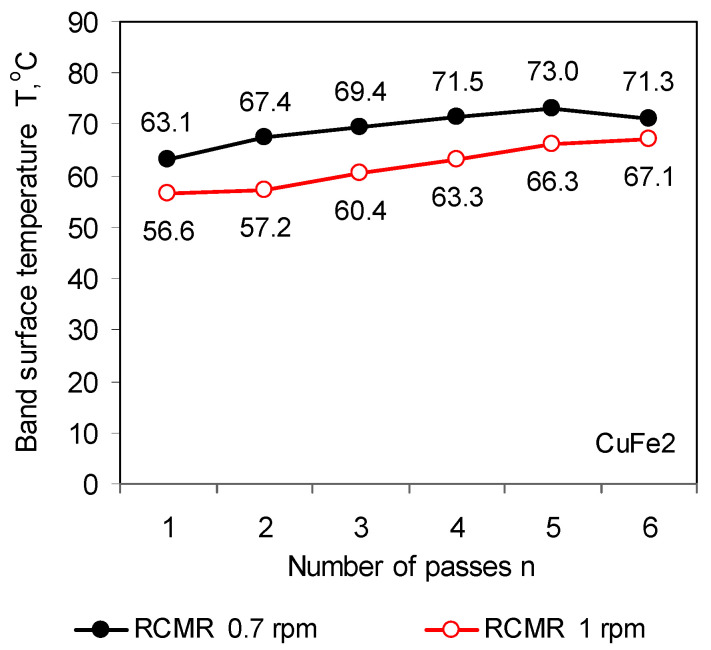
Band surface temperature in the RCMR rolling process of the CuFe2 alloy with rolling speeds ω = 0.7 rpm and 1 rpm.

The lower rolling speed in the RCMR rolling process causes a significant increase in the strip surface temperature (Figure 12). This is due to the increased intensity of the strand deformation. At a lower rolling speed, the rolls perform a greater number of axial motion cycles along the length of the rolling gap, assuming that other rolling parameters, frequency f and roll deflection amplitude A, are constant. Of course, this is only one of many possibilities of increasing the deformation intensity as a result of changing the kinematic parameters of rolling in the RCMR process.

During plastic deformation, the macroscopic behavior of the material is the result of changes in its microstructure. The process of plastic deformation always causes a change in the temperature field in the deformed material, which is a macroscopic response of the phenomena, occurring at the level of its microstructure. The analysis of the results of thermo-vision tests and the development of the energy balance of such complex processes can be helpful in identifying the mechanisms responsible for the decrease in strain work, and an increase in the plasticity of the materials.

## 6. Conclusions

The presented experimental studies were aimed at determining the effective conditions for controlled interference in the process of plastic flow in the new method of rolling. The conducted research showed the significant research potential of the method. Relationships between strain components and structural effects responsible for the rolling pressure drops and deformation softening of metals were determined. The new method makes it possible to apply a large cumulative deformation in a single pass and to deform samples of “unlimited length”. In the analysis of mechanical and structural phenomena accompanying the rolling process, it is also necessary to use numerical modeling tools [27].

The method is developing and has great application potential, but it requires the solution of difficult construction problems related to the kinematics of tool movement and the effectiveness of the impact on the deformed material. Implementation of the new rolling method is particularly important due to the dominant share of these rolling processes in plastic manufacturing technology, mass production, and the large variety of the range of products. Even a limited application of the method, e.g., for initial processing, can shorten the production cycle by reducing the number of unit operations or rolling stands in the technological line. The expected benefits of using the new method of rolling include, above all, obtaining a large cumulative deformation, reducing the number of passes, lowering rolling pressures, lowering the unit work of plastic deformation (energy-saving process) and obtaining the desired initial metal structure.

Due to the occurrence of large, cyclically variable shear stresses, effectively penetrating the band at height, the method can be used in recycling processes for layered composite materials. Rolling tests carried out on strips made of layered laminates gave very promising results. Laminate layers were effectively separated. It is also possible to recycle rubber-based materials reinforced with steel cords, e.g., conveyor belts, car tires, etc. The rolling method allows for effective and quick separation of components.

It is justified to conduct further laboratory tests and technological trials in order to fully recognize the possibilities of the method, which is currently at the fourth level of technological readiness, i.e., TRL (Technology Readiness Level).

The conducted experimental studies allowed the formulation of the following conclusions:

1. The selection of too extreme kinematic rolling parameters in the RCMR process, and above all the high amplitude and frequency of axial roll movement, may result in band rotation, which affects the amount of the set actual cumulative deformation and causes an undesirable effect of distortion of the cross-section of the band.
2. In the RCMR process, we obtain much larger deformations in a single pass compared to conventional rolling.
3. Zones of intensive local plastic deformation in the RCMR process are formed at the contact surface with the upper and lower cylinder and, in subsequent culverts, migrate from the surface towards the middle of the band, covering an increasing volume. This has a positive effect on the uniformity of deformations in the volume of the rolled band.
4. Forcing additional form deformation in the RCMR process has significant effects in the form of a decrease in rolling pressure forces compared to the conventional process. This effect is independent of the type of material and the initial state after heat treatment.
5. Thermographic analysis showed an intensive increase in band surface temperature in the RCMR process compared to conventional rolling.
6. The rolling method can become an interesting complement to the existing methods used in large plastic deformations. It also has a large application capacity in industrial metal deformation and recycling processes.

## Data Availability

The data presented in this study are available on request from the author.
